# Newly Discovered Action of HpTx3 from Venom of *Heteropoda venatoria* on Na_v_1.7 and Its Pharmacological Implications in Analgesia

**DOI:** 10.3390/toxins11120680

**Published:** 2019-11-20

**Authors:** Xinzhou Wu, Zhouquan Wang, Yu Chen, Dehong Xu, Peng Zhang, Xianchun Wang

**Affiliations:** Key Laboratory of Protein Chemistry and Developmental Biology of Ministry of Education, College of Life Sciences, Hunan Normal University, Changsha 410081, China

**Keywords:** HpTx3, Na_v_1.7, inhibition, selectivity, analgesia, mouse pain model, *Heteropoda venatoria*

## Abstract

It has been reported that Heteropodatoxin3 (HpTx3), a peptidic neurotoxin purified from the venom of the spider species *Heteropoda venatoria,* could inhibit K_v_4.2 channels. Our present study newly found that HpTx3 also has potent and selective inhibitory action on Na_v_1.7, with an IC_50_ of 135.61 ± 12.98 nM. Without effect on the current–voltage (I-V) relationship of Na_v_1.7, HpTx3 made minor alternation in the voltage-dependence of activation and steady-state inactivation of Na_v_1.7 (4.15 mV and 7.29 mV, respectively) by interacting with the extracellular S3–S4 loop (S3b–S4 sequence) in domain II and the domain IV of the Na_v_ channel subtype, showing the characteristics of both pore blocker and gate modifier toxin. During the interaction of HpTx3 with the S3b–S4 sequence of Na_v_1.7, the amino acid residue D in the sequence played a key role. When administered intraperitoneally or intramuscularly, HpTx3 displayed potent analgesic activity in a dose-dependent manner in different mouse pain models induced by formalin, acetic acid, complete Freund’s adjuvant, hot plate, or spared nerve injury, demonstrating that acute, inflammatory, and neuropathic pains were all effectively inhibited by the toxin. In most cases HpTx3 at doses of ≥ 1mg/kg could produce the analgesic effect comparable to that of 1 mg/kg morphine. These results suggest that HpTx3 not only can be used as a molecular probe to investigate ion channel function and pain mechanism, but also has potential in the development of the drugs that treat the Na_v_1.7 channel-related pain.

## 1. Introduction

The voltage-gated sodium channel (Na_v_) in mammalian cells is a multiple transmembrane protein that can be activated by voltage and then permits the Na^+^ to enter the cell. To date, a total of nine kinds of Na_v_ subtypes have been found and named Na_v_1.1–1.9 [1,2]. A Na_v_ channel consists of a pore-forming α subunit and one or two β subunits and only the α subunit is required for function. The α subunit is composed of four homologous domains. Each domain contains six transmembrane helices (S1–S6) and a certain number of extra- and intra-cellular loops connect these transmembrane helices. S1–S4 have been shown to sense voltage and S5–S6 to construct the central pore [3]. Although the Na_v_ channel has diverse in vivo distribution profiles and biological functions, its fundamental function is to produce and transmit currents in cell membrane by permitting the flow of ions [4]. The disorder of a Na_v_ channel often leads to many kinds of diseases, collectively named ion-channel diseases. For example, Na_v_1.7 dysfunction is closely involved in pain [5]. Pain is a protective sensation that serves to warn us of impending harm and makes us withdraw from and subsequently avoid injurious situations. However, abnormal pain will deteriorate patients’ life quality, expend medical resources, or even threat patients’ lives. Na_v_1.7 is primarily expressed in the peripheral nervous system and participates in the linkage of stimuli and pain signaling pathway. Sensory neurons in the peripheral nervous system can produce action potential when the stimulation is applied, and Na_v_1.7 plays an important role in initiating such action potentials [5,6]. It has been well-established that Na_v_1.7 is highly expressed in dorsal root ganglia neurons and its mutations induce genetic pain and painless disorders, which makes Na_v_1.7 one of the most promising targets for pain control [7,8,9]. For example, gain-of-function mutation in the *SCN9A* gene that encodes the α subunit of Na_v_1.7 caused severe episodic pain, whereas loss-of-function mutations in *SCN9A* resulted in insensitivity to pain [10]. Furthermore, many reports indicate that the gating modulators and pore blockers of Na_v_1.7 can affect the sensitivity of a patient to pain [11]. Therefore, the agents targeting Na_v_1.7 can help patient relief or treat the Na_v_1.7-related pain syndromes [5].

Up to date, a batch of small molecules that target Na_v_ channels are well established to attenuate the pain in humans, such as opioids [12] and tetrodotoxin (TTX), a well-known blocker of Na_v_ channels [13]. Nevertheless, due to their intrinsic limitations, such as lack of specificity and/or pronounced side-effects, the potential of these small molecules in the therapeutic development is significantly weakened, which promotes development of the drugs with higher efficiency and less side effects from natural resources. In recent decades, the proteinaceous toxins with an analgesic effect from the venomous spiders have attracted the attention of the relevant scientific researchers [7,14]. Spider venom produced by venom glands can help spiders resist the natural enemies and capture preys. The venom is a highly complex mixture composed of small molecules, peptides, proteins, etc. Peptides are often of high level in the spider venom and many of them can specifically act on ion channels, including Na_v_1.7 [15,16], and thus have potential in the development of drugs that treat Na_v_ 1.7 subtype-related diseases.

In this paper, we isolated a peptide toxin, named Heteropodatoxin3 (HpTx3) [17], from the venom of the spider *Heteropoda venatoria* and proved that HpTx3 has high potency and selectivity against Na_v_1.7. Using multiple rodent pain models, HpTx3 was demonstrated to have a powerful analgesic effect.

## 2. Results

### 2.1. Preparation and Identification of HpTx3

**Figure 1:**
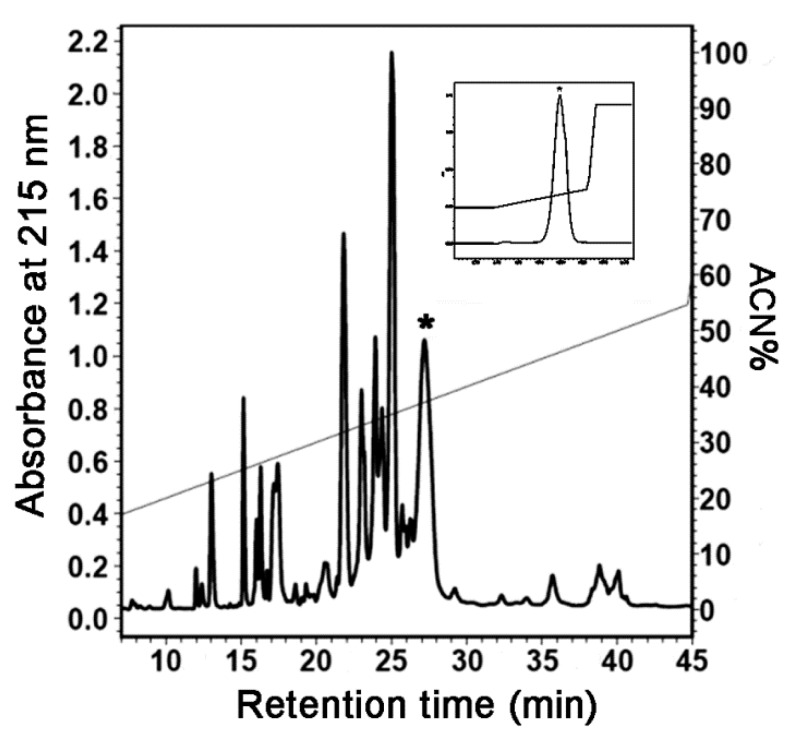
RP-HPLC chromatograms of the *Heteropoda venatoria* venom. The asterisk (*) indicates the peak of interest and the inset shows its further purification by analytical RP-HPLC.

**Figure 2:**
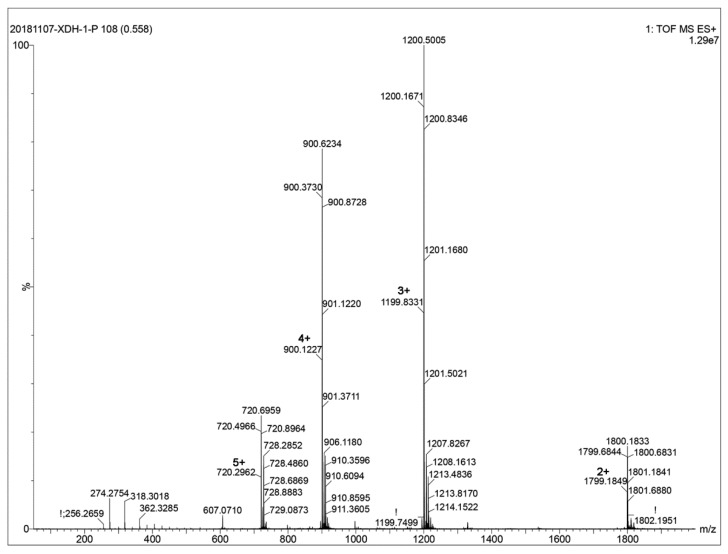
Determination of the molecular weight of Heteropodatoxin3 (HpTx3) by mass spectrometry.

After the venom was electrically collected from the spider species *Heteropoda venatoria* and was subjected to semipreparative RP-HPLC, it was separated into more than ten primary chromatographic peaks (Figure 1). The peak marked with an asterisk corresponded to that containing HpTx3 in the RP-HPLC chromatogram of the *Heteropoda venatoria* venom reported by Sanguinetti et al. [17], and was collected and further purified with an analytical C18 column, which gave a single symmetric peak (inset in Figure 1), suggesting that the sample was purified to homogeneity. The mass spectrometric analysis indicated that the venom component existed primarily in the form of multiple-charge ions with 2, 3, 4, and 5 positive charges, respectively (Figure 2). From the *m*/*z* values of its ion peaks, the monoisotopic molecular weight of the component was calculated to be 3596.46 (average molecular weight 3599), which was consistent with the theoretical monoisotopic molecular weight of the Heteropodatoxin3 (HpTx3) with an amide on its C-terminal [17]. In view of the fact that the acquired venom component and the reported HpTx3 had the corresponding peak position in RP-HPLC chromatogram and the same molecular weight, the component we purified from the *Heteropoda venatoria* venom was identified as peptide neurotoxin HpTx3.

### 2.2. Effects of HpTx3 on Na_v_ Channel Subtypes

**Figure 3:**
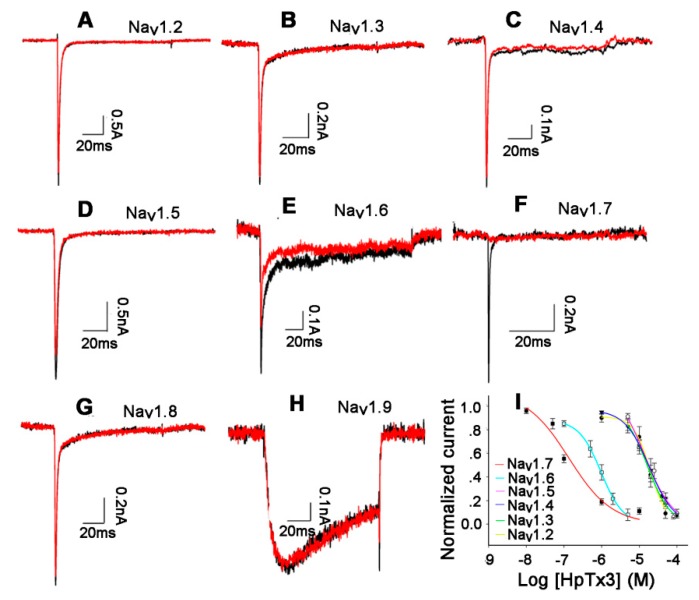
Effects of HpTx3 on Na_v_ channel subtypes. (**A**–**H**) Representative current traces of Na_v_ subtypes (Na_v_1.2–1.9, respectively) before (black) and after (red) addition of 1 μM (Na_v_1.2–1.7) or 10 μM (Na_v_1.8–1.9) HpTx3 (n = 4–6); (**I**) concentration-response curves for the inhibition of Na_v_ subtypes by HpTx3 (n = 4–6).

By sequence alignment, we found that the sequence of HpTx3 has high identity with those of some Na_v_ channel peptide toxins in the NaSpTx family 3 [7,18] (see Discussion), which suggested that HpTx3 might have an inhibitory effect on Na_v_ channels. In order to investigate the Na_v_ channel-targeted activity and the subtype selectivity of HpTx3, the effects of HpTx3 on mammal Na_v_1.2–1.9 expressed in HEK293T or ND7/23 cells were detected. The results demonstrated that 1 μM HpTx3 inhibited the Na_v_1.7 currents completely, Na_v_1.6 currents by 46.0%, and Na_v_1.5 currents by 12.8%, without effects on Na_v_1.2, Na_v_1.3, and Na_v_1.4; even 10 μM HpTx3 did not affect the currents of Na_v_1.8 and Na_v_1.9 (Figure 3A–H). Comparatively, HpTx3 most potently inhibited Na_v_1.7 (IC_50_ 135.61 ± 12.98 nM), with 145-fold selectivity over Na_v_1.2 (IC_50_ 19.71 ± 0.49 μM), 104-fold selectivity over Na_v_1.3 (IC_50_ 14.11 ± 2.5 μM), 136-fold selectivity over Na_v_1.4 (IC_50_ 18.5 ± 0.21 μM), 122-fold selectivity over Na_v_1.5 (IC_50_ 16.51 ± 0.85 μM), 7-fold selectivity over Nav1.6 (IC_50_ 972.10 ± 1.50 nM), and even greater selectivity over Na_v_1.8 and Na_v_1.9 whose IC_50_ values were not determined in view of the fact that 10 μM HpTx3 did not significantly affect their currents. These data demonstrated that the effect of HpTx3 on Na_v_ channels has obvious subtype selectivity (Figure 3I).

### 2.3. Effects of HpTx3 on the Voltage-Dependence of Na_v_1.7 Activation and Inactivation 

**Figure 4:**
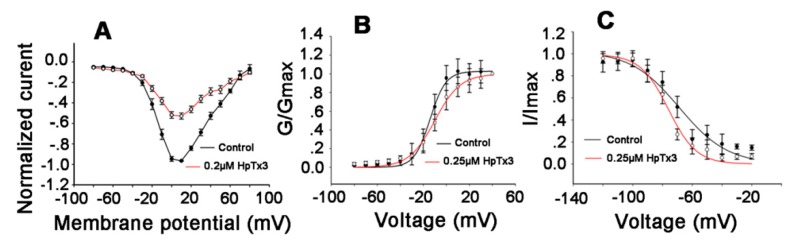
Effects of HpTx3 on the voltage-dependence of Na_v_1.7 activation and steady-state inactivation. (**A**) Current–voltage (I-V) curve for Na_v_1.7 before (black) and after (red) application of HpTx3 (n = 6); (**B**) steady-state activation curves of Na_v_1.7 before (black) and after (red) application of HpTx3 (n = 6); (**C**) steady-state inactivation curves of Na_v_1.7 before (black) and after (red) application of HpTx3 (n = 6). Data are shown as mean ± SD.

When the current–voltage (I–V) relationship for Na_v_1.7 affected by HpTx3 was investigated, the results indicated that HpTx3 (0.20 µM) voltage-dependently decreased the currents of Na_v_1.7 at the voltages ranging from −40 mV to + 70 mV, but did not significantly alter the threshold value of initial activation voltage (−40 mV), the maximum activation voltage (+10 mV) and the reversal voltage (+70 mV) of the Na_v_ channel subtype (Figure 4A), suggesting that the application of the toxin did not change the ion selectivity of Na_v_1.7 channel in the tested depolarizing voltages. However, as shown in Figure 4B, compared with the control, HpTx3 (0.25 µM) shifted the steady-state activation curve of Na_v_1.7 to the right (more depolarized membrane voltage) by about 4.15 mV, with half-activation voltages before and after the application of HpTx3 being −14.20 ± 1.27 mV and −10.05 ± 0.96 mV, respectively. The analysis of inactivation kinetics of the Na_v_1.7 showed that the half-inactivation voltages before and after the application of 0.25 µM HpTx3 were −68.71 ± 0.58 mV and −76 ± 0.78 mV, respectively, shifting the steady-state inactivation curve to the left (more polarized membrane voltage) by 7.29 mV (Figure 4C), indicating that HpTx3 facilitated the steady-state inactivation of the Na_v_1.7 channel subtype to a certain degree.

### 2.4. Action Sites of HpTx3 on Na_v_1.7

**Figure 5:**
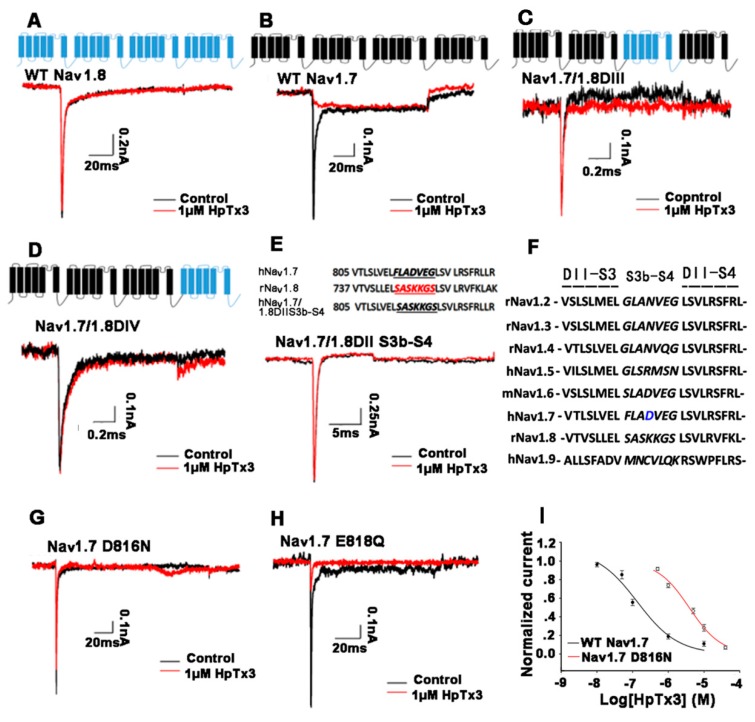
Effects of HpTx3 on the currents of chimeras and single mutants. (**A**,**B**) Membrane topology of wild type Na_v_1.7 and Na_v_1.8, and the effect of HpTx3 on their currents; (**C**,**D**) membrane topology of chimeras Na_v_1.7/1.8DIII and Na_v_1.7/1.8DIV, and the effect of HpTx3 on their currents; (**E**) construction of Na_v_1.7/1.8DIIS3b–S4 and the effect of HpTx3 on its current; (**F**) alignment of the S3b–S4 sequences of Na_v_ subtypes. The key amino acid residue is highlighted with blue color; (**G**,**H**) effect of HpTx3 on the currents of Na_v_1.7 D816N and Na_v_1.7 E818Q; (**I**) concentration-response curves of HpTx3 on wild type Na_v_1.7 and Na_v_1.7 D816N. n = 6.

In order to identify the action sites of HpTx3 on Na_v_1.7, we inserted DIIS3b–S4, DIII, and DIV sequences of Na_v_1.8, on which 10 μM HpTx3 had no inhibitory effect (Figure 3G), into the Na_v_1.7 to replace the counterpart sequences and thus prepared three chimeras named Na_v_1.7/1.8DIIS3b–S4, Na_v_1.7/1.8DIII, and Na_v_1.7/1.8DIV, respectively (Figure 5A–E), followed by patch clamp analysis to detect the effects of HpTx3 on the currents of the resulting chimeras expressed in HEK293T cells. The analytical results indicated that 1 μM HpTx3 showed no significant effects on the currents of Na_v_1.7/1.8DIV (Figure 5D) and Na_v_1.7/1.8DIIS3b–S4 (Figure 5E), and the current of the Na_v_1.7/1.8DIII was enhanced by the toxin at the same concentration (Figure 5C). These results demonstrated that the DIV and DIIS3b–S4, an extracellular loop of DII, of the Na_v_1.7 are involved in the interaction between HpTx3 and Na_v_1.7; the mechanism of action of HpTx3 was speculated to be at least partially similar to that of site 4 toxins that use DIIS3b–S4 as a key action site [19,20]. In order to further identify the key residues in the DIIS3b–S4 sequence of Na_v_1.7, we first compared the DIIS3b–S4 sequences of Na_v_1 subtypes (Figure 5F). As shown in Figure 5F, the D/E and E/Q may be the amino acids of potential binding sites. We focused on comparing the S3b–S4 sequence of Na_v_1.7 with that of Na_v_1.4, on which 1 µM HpTx3 showed no inhibitory effect (Figure 3C), and found that only two amino acid residues are different: D in Na_v_1.7 DIIS3b–S4 compared to N in Na_v_1.4 DIIS3b–S4 and E in Na_v_1.7 DIIS3b–S4 compared to Q in Na_v_1.4 DIIS3b–S4. Therefore, we mutated the two acidic amino acid residues of Na_v_1.7 DIIS3b–S4 into neutral ones, D into N (Na_v_1.7 D816N) and E into Q (Na_v_1.7 E818Q). Patch clamp analysis showed that 1 μM HpTx3, which could inhibit the current of wild type Na_v_1.7 completely (Figure 3F), inhibited the current of Na_v_1.7 D816N by only 23.7% (Figure 5G), with an IC_50_ value of 3.13 ± 2.6 μM, compared to 135.61 ± 12.98 nM for wild type Na_v_1.7 (Figure 5I), showing a 23-fold difference. On the contrary, 1 μM HpTx3 inhibited the current of the Na_v_1.7 E818Q by about 80% (Figure 5H). These data demonstrated that, during the HpTx3 interaction with Na_v_1.7, residue D816 in the DIIS3b–S4 sequence of Na_v_1.7 plays a crucial role, and E818 only plays a minor role.

### 2.5. Analgesic Effects of HpTx3 in Mouse Pain Models

In order to probe into the pharmacological implications of HpTx3 in analgesia, we used different mouse pain models, including those induced by formalin, acetic acid, complete Freund’s adjuvant, hot plate, and spared nerve injury, to detect the effects of the toxin on the pain-like behaviors of the mice. The pain models could be classified into acute inflammation models induced by formalin and acetic acid, chronic inflammation pain model induced by complete Freund’s adjuvant, acute thermal pain model induced by hot plate, and chronic neuropathic pain model induced by spared nerve injury.

#### 2.5.1. Formalin Model Test

**Figure 6:**
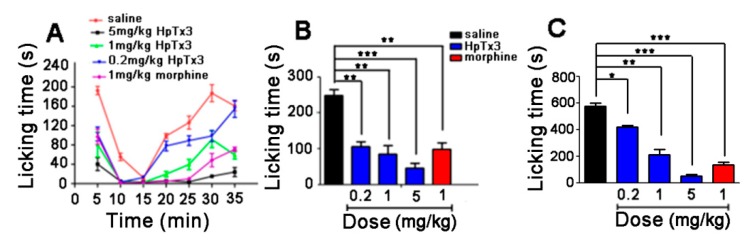
Effects of HpTx3 on the paw licking time of the mice in formalin-induce pain model. (**A**) Licking time at different time points after injection; (**B**) total licking time in phase I after injection; (**C**) total licking time in phase II after injection. The data are shown as mean ± SD, n = 8. * *p* < 0.05, ** *p* < 0.01, *** *p* < 0.001.

In the formalin-induced mouse pain model, nociceptive behaviors such as paw-licking, swinging legs, and retractable legs in mice were induced by subcutaneous injection of formalin. The effect of HpTx3 on the formalin-induced nociceptive behaviors was assessed by comparing the times in seconds of paw licking in mice injected intramuscular with 100 μL 0.9% saline (control), morphine (1 mg/kg, positive control), or HpTx3 (0.2, 1, and 5 mg/kg). Paw licking times were tallied in 5-min time bins over 35 min following formalin injection (Figure 6). As shown in Figure 6A, the nociceptive reaction included phase I (0–10 min after formalin injection) and phase II (15–35 min after formalin injection), which were mainly caused by the direct activation of nociceptive neurons and the formalin-induced inflammation, respectively [21,22]. Compared with the control, HpTx3 shortened the paw licking times in both of the two phases, indicating that the toxin could attenuate the nociceptive behaviors caused by both nociceptive neuron activation and inflammation. In phase I, the total paw licking times of the mice in the control group were 248 s, while those of the mice in the groups treated with 0.2, 1, and 5 mg/kg HpTx3 were 106 s, 85.3 s, and 45.3 s, shortening the paw licking times by 57.26% (*p* < 0.01), 65.60% (*p* < 0.01), and 81.73% (*p* < 0.001), respectively (Figure 6B). In phase II, the total paw licking times of the mice in the control group were 572.7 s, while those of the mice in the groups treated with 0.2, 1, and 5 mg/kg HpTx3 were 424.3 s, 210.3 s, and 49 s, decreasing the times by 25.91% (*p* < 0.05), 63.27% (*p* < 0.01), and 91.44% (*p* < 0.001), respectively. These data demonstrated that HpTx3 at the tested concentrations could significantly attenuate the nociceptive behaviors induced by formalin and the analgesic effects of HpTx3 in both of the two phases were dose-dependent. In addition, the total paw licking times of the mice in the group treated with 1 mg/kg morphine were 99 s in phase I and 134.3 s in phase II, respectively. Comparison of the licking times showed that the paw licking times after injection of 0.2, 1 and 5 mg/kg HpTx3 in phase I, and 1 and 5 mg/kg HpTx3 in phase II were not significantly different from those after injection of 1 mg/kg morphine (*p* > 0.05), suggesting their comparable analgesic effects.

#### 2.5.2. Acetic Acid-Induced Writhing Model Test

**Figure 7:**
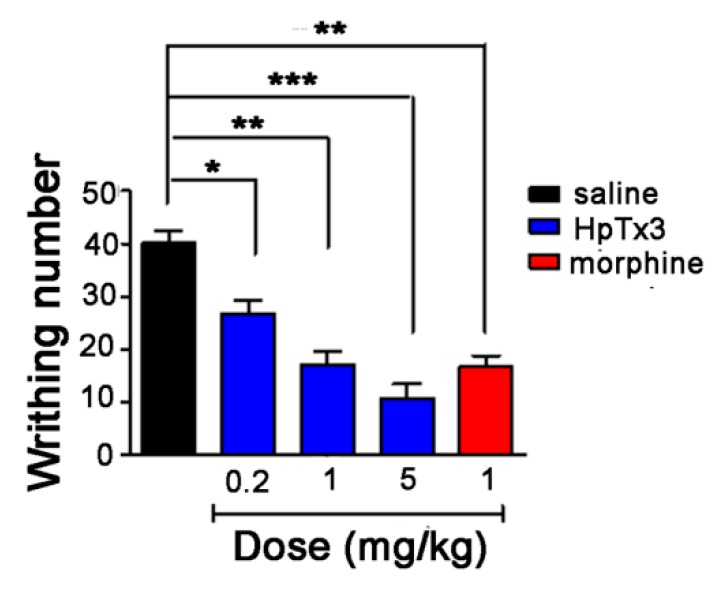
Effects of HpTx3 on the abdominal writhing number of the mice in acetic acid-induced writhing model. The data are shown as mean ± SD, n = 8. * *p* < 0.05, ** *p* < 0.01, *** *p* < 0.001.

In the acetic acid-induced writhing model, the nociceptive behaviors were induced, including writhing, body stretching, abdomen sticking to the ground, etc. As shown in Figure 7, the average writhing number of the mice in the control group over 30 min was 40.3, while those of the mice in the test groups treated with HpTx3 at doses of 0.2, 1, and 5 mg/kg were 26.8, 17, and 10.8, decreasing the writhing number by 33.54% (*p* < 0.05), 57.76% (*p* < 0.01), and 73.29% (*p* < 0.001), respectively. These findings indicated that HpTx3 significantly decreased the number of abdominal writhing of the mice in a dose-dependent manner. The average abdominal writhing number of the mice intraperitoneally injected with morphine at a dose of 1 mg/kg was 16.8, compared to 17 for 1 mg/kg HpTx3 (*p* > 0.05) and 10.8 for 5 mg/kg HpTx3 (*p* > 0.05), respectively, demonstrating that in the acetic acid-induced writhing model 1 and 5 mg/kg HpTx3 produced analgesic action comparable to that of 1 mg/kg morphine (*p* > 0.05).

#### 2.5.3. Complete Freund’s Adjuvant (CFA) Model Test 

**Figure 8:**
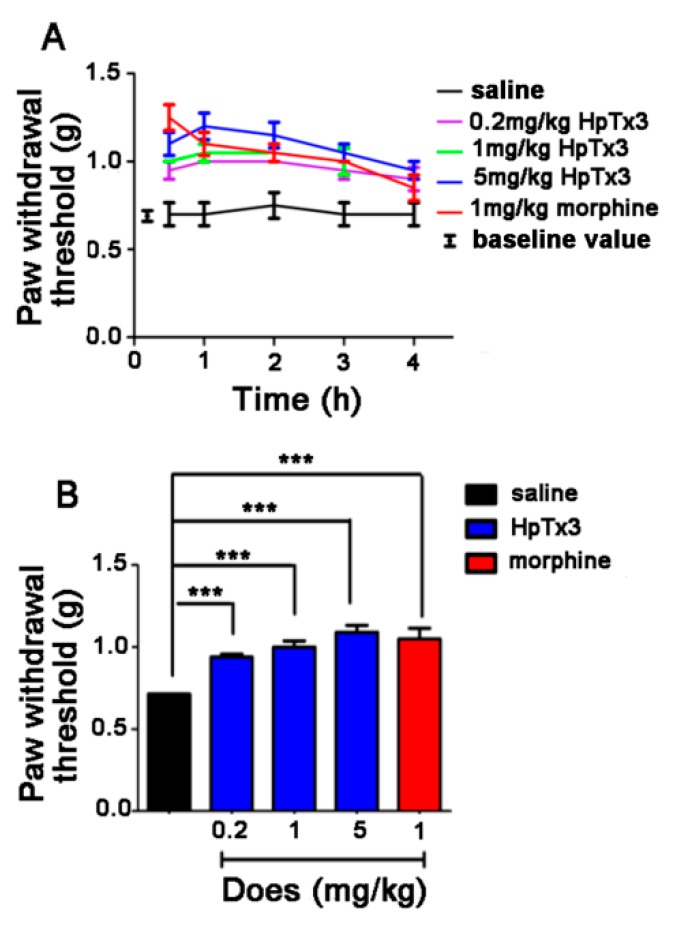
Effect of HpTx3 on the inflammatory pain-like behaviors in mice in complete Freund’ s adjuvant (FCA) pain model. (**A**) Paw withdrawal threshold detected at different time points after FCA injection; (**B**) comparison of average paw withdrawal threshold after injection with different concentrations of toxin or morphine. The data are shown as mean ± SD, n = 8. *** *p* < 0.001.

Twenty-four hours after local inflammation in mice was induced by subcubaneous injection of complete Freund’s adjuvant (CFA) into the plantar surface of the right hind paw, it led to nociceptive behaviors such as paw-licking and flinching. All the mice developed mechanical hyperalgesia in the inflamed hind paw, with an average withdrawal threshold of 0.70 ± 0.20 g compared to 1.85 ± 0.28 g before CFA injection. As shown in Figure 8A, intramuscular administration of 0.2, 1 and 5 mg/kg HpTx3 significantly increased the paw withdrawal thresholds to 0.94, 1 and 1.09 g, compared to 0.71 g of the control (*p* < 0.001), respectively, displaying an obvious dose-dependent analgesic effect. The strongest analgesic action of HpTx3 at the three different concentrations occurred at 1 h after HpTx3 injection. After this time point, the toxin still had a strong analgesic effect within the time range of the test, although having a decreasing tendency. Different from HpTx3, morphine, as a positive control in the present test, displayed the strongest analgesic effect at 0.5 h after injection, indicating that morphine exerted the analgesic action faster than HpTx3 in such a model. Figure 8B shows that HpTx3 increased the paw withdrawal threshold in a dose-dependent manner, and HpTx3 at all the used doses (0.2, 1, and 5 mg/kg) produced an analgesic effect close to that (paw withdrawal threshold 1.05 g) of 1 mg/kg morphine (*p* > 0.05).

#### 2.5.4. Hot Plate Pain Model Test

**Figure 9:**
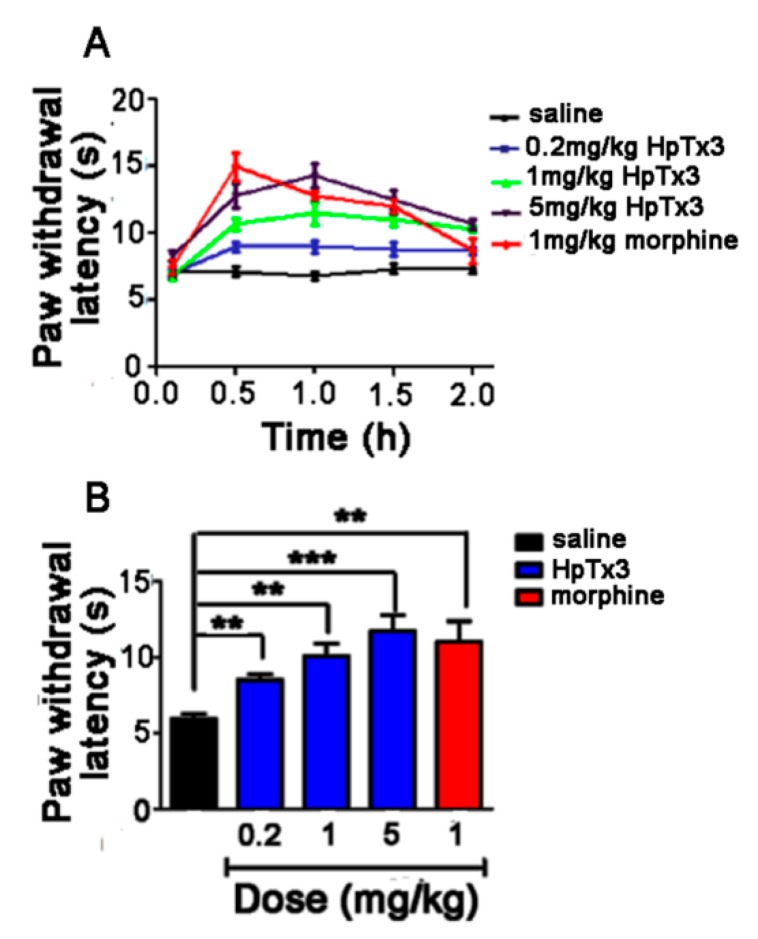
Effect of HpTx3 on paw withdrawal latency of the mice in hot plate pain model. (**A**) Paw withdrawal latency detected at different time points after injection; (**B**) Comparison of average paw withdrawal latency after injection with different concentrations of toxin or morphine. The data are shown as mean ± SD, n = 8. ** *p* < 0.01, *** *p* < 0.001.

When the temperature of the hot plate was set at 55 ± 1 °C, the mice showed obvious nociceptive behaviors including paw-licking and paw lift. The paw withdrawal latency of the mice placed on the plate was determined in seconds at 0.5, 1.0, 1.5, and 2.0 h after injection. The paw withdrawal latency of the control (injection of 0.9% saline) remained relatively stable at all the time points, with an average paw withdrawal latency of 5.93 s. Intraperitoneal injection of HpTx3 increased the paw withdrawal latency at all four time points after injection, showing the most analgesic effect at 1.0 h (at doses of 1 and 5 mg/kg) as well as 0.5 h (at a dose of 0.2 mg/kg) (Figure 9A), while morphine displayed the most analgesic effect at 0.5 h after injection. The average paw withdrawal latency of the mice injected with HpTx3 at doses of 0.2, 1, and 5 mg/kg were 8.5, 10.07, and 11.7 s, which, compared with the control, increased paw withdrawal latency by 43.33% (*p* < 0.01), 69.81% (*p* < 0.01), and 97.3% (*p* < 0.001), respectively (Figure 9B). These results indicated that HpTx3 significantly raised the thermal pain threshold of the mice in a dose-dependent way. Furthermore, statistical analysis showed that the potency of 1 and 5 mg/kg HpTx3 to inhibit thermal pain-like behaviors was comparable to that (average paw withdrawal latency 11.03 s) of 1 mg/kg morphine (*p* > 0.05) (Figure 9B).

#### 2.5.5. Spared Nerve Injury (SNI) Model Test

**Figure 10:**
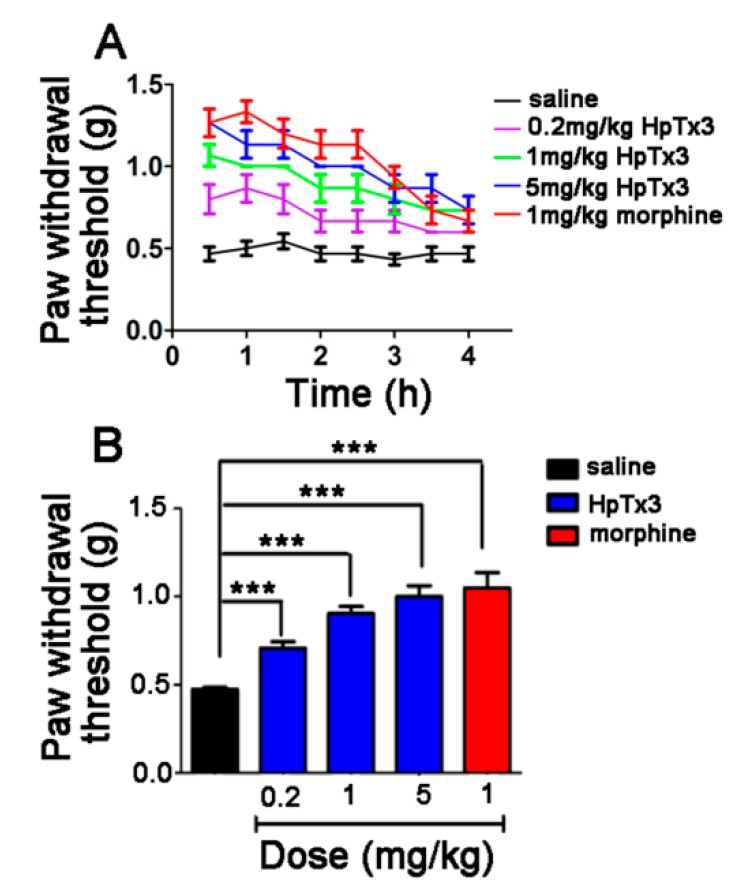
Effect of HpTx3 on paw withdrawal threshold of the mice following mechanical stimuli to the paw. The determination of paw withdrawal thresholds was made 18 days after the surgery. (**A**) Paw withdrawal thresholds detected at different time points after injection; (**B**) comparison of average paw withdrawal threshold after injection with different concentrations of toxin or morphine. The data are shown as mean ± SD, n = 8. *** *p* < 0.001.

Observation showed that, three days after the SNI surgery, the mice developed mechanical hyperalgesia that lasted for about one month at the ipsilateral paw, showing obvious nociceptive behaviors such as flinching and paw lift. We began to determine the paw withdrawal thresholds 18 days after the surgery. On the 18th day, the average paw withdrawal threshold in mice was 0.46 ± 0.10 g compared to 1.85 ± 0.28 g before the surgery, indicating that the SNI model was prepared successfully. As shown in Figure 10A, 1 and 5 mg/kg HpTx3 showed their highest analgesic activity at 0.5 h after injection, and 5 mg/kg HpTx3 produced stronger analgesic action than 1 mg/kg morphine at the time points after 3 h, indicating that 5 mg/kg HpTx3 could produce an analgesic effect in an even longer time. The average paw withdrawal threshold of the mice in control was 0.47 g, and injection of 0.2, 1, and 5 mg/kg HpTx3 increased the average threshold to 0.71, 0.9, and 1 g, increased by 51.06%, 91.49%, and 112.77%, respectively (*p* < 0.001, Figure 10B). In addition, 1 mg/kg morphine led to an average paw withdrawal threshold of 1.05 g, showing a comparable analgesic effect to 1 and 5 mg/kg HpTx3 (*p* > 0.05).

### 2.6. In Vivo Toxicity and the Effect of HpTx3 on the hERG Channel

For detecting the potential in vivo toxicity of HpTx3, we chose eight C57BL/6 mice and intraperitoneally injected each of them with 20 mg/kg HpTx3. This dose was four-fold greater than the maximum dose (5 mg/kg) and 100-fold greater than the minimum dose (0.2 mg/kg) that were used in analgesic experiments to produce analgesic effects. The results showed that within 5 min after the injection of HpTx3 at a dose of 20 mg/kg, the mice exhibited some discomfort symptoms such as body curling and writhing; after 5 min, the symptoms became gradually weaker and disappeared completely after 10 min. 

**Figure 11:**
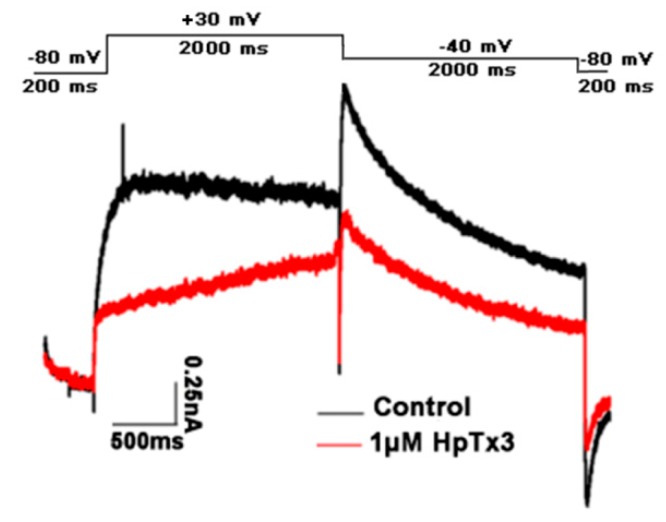
Effect of HpTx3 on the currents of the hERG channel (n = 3).

In order to detect the effect of HpTx3 on the hERG channel, we expressed the channel in HEK293T cells, followed by analysis with the whole cell patch-clamp technique. The results showed that 1 µM HpTx3 inhibited about 40% of the hERG channel current (Figure 11), suggesting that HpTx3 might have some potential cardiotoxicity. In view of the above results that even 20 mg/kg HpTx3 only caused weak and short-period adverse effects on the mice, whether the HpTx3 applied at the analgesic doses (0.2–5 mg/kg) causes adverse effects on the heart needs further investigation, and the cardiotoxicity, if indeed present, could be alleviated or eliminated by structure modification such as mutation. 

## 3. Discussion

**Figure 12:**
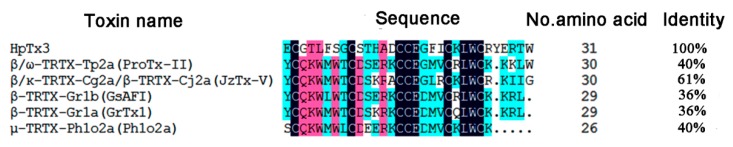
Sequence alignment of HpTx3 and some toxins in the NaSpTx family 3.

In 1997, Sanguinetti et al. [17] isolated three new peptide toxins from the venom of the spider species *Heteropoda venatoria*, named Heteropodatoxin 1 to Heteropodatoxin 3 (HpTx1 to HpTx3). These three toxins are structurally similar and have amides on their C-termini. The conserved arrangement of the cysteine residues in HpTxs indicated that they contain an inhibitor cystine knot (ICK) motif. Electrophysiological experiments indicated that HpTxs can block K_v_4 channels and the effects of the HpTxs, particularly HpTx 2 and HpTx3, on K_v_4.2 channel subtype have been extensively characterized [17,23,24,25,26,27,28,29,30]. Our previous research found that the venom of *Heteropoda venatoria* has potent inhibitory effects on voltage-gated Na^+^ channels in *Periplaneta americana* dorsal unpaired medium (DUM) neurons and rat dorsal root ganglion (DRG) neurons, indicating that the venom not only is rich in K^+^ channel blocker neurotoxins, but also contains diverse Na^+^ channel inhibitors [31]. These findings aroused our interest. By sequence alignment, we found that the sequence of HpTx3 has a high identity with those of some Na_v_1.7 potential analgesic peptide toxins from the Na_v_-targeting spider toxin family 3 (NaSpTx family 3) [7,18] (Figure 12), which suggested that HpTx3 might have an inhibitory effect on the Na_v_1.7 channel. Our present research isolated the HpTx3 from the venom of the spider species *Heteropoda venatoria* and experimentally demonstrated that the peptide toxin has a selective inhibitory effect on the Na_v_1.7 channel.

It is generally accepted that the likely promising target for therapeutic treatment to fight pain and avoid central side-effects is the neurons located in the periphery DRGs that convey pain from the skin and tendons to the central nervous system [7]. The Na_v_ 1.7 channel subtype is considered as one of the most promising antinociceptive targets for analgesic drugs because it is abundantly present in the peripheral DRG neurons and has facilitated permeability to high molecular weight drugs [7]. Na_v_1.7-targeting peptide toxins from spider venoms have been proposed as the candidates to replace opioids to treat pain, as the peptide toxins have advantages such as specific interaction with ion channels, easy expression, chemical synthesis, etc. [7]. Our present research found a new peptide candidate, HpTx3, which might be used to replace opioids to treat pain. HpTx3 is composed of 31 amino acids and has three disulfide bonds arranged in an inhibitor cystine knot (ICK) motif. This toxin was demonstrated to potently inhibit Nav1.7 (IC_50_ 135.61 ± 12.98 nM), with at least seven-fold selectivity over other tested Na_v_ channel subtypes (Figure 3). It is commonly accepted that the spider toxins targeting the Na_v_1.7 subtype with an IC_50_ less than 500 nM are considered as analgesic toxin inhibitors [32]. Therefore, HpTx3 can be considered as an analgesic toxin inhibitor with high selectivity for the Na_v_1.7. 

In order to further understand the actions of HpTx3 on the Na_v_1.7 channel, we examined the effect of the toxin on the current–voltage (I–V) relationship of the Na_v_ channel subtype. The results indicated that HpTx3 decreased the peak current of Na_v_1.7, but did not significantly change the initial activation voltage, the activation voltage of the maximum Na_v_1.7 current, and the ion selectivity of the channel at depolarizing voltages ranging from −80 mV to +80 mV. When the conductance–voltage (G–V) relationship and the steady-state inactivation of Na_v_1.7 before and after HpTx3 treatment were investigated, it was found that HpTx3 induced minor alternation in the voltage-dependence of Na_v_1.7, shifting the activation curve to the right by 4.15 mV and the steady-state inactivation curve to the left by 7.29 mV (Figure 4C). These results suggested that the molecular mechanism of HpTx3 on Na_v_1.7 is different from that of ProTx-II that shifts Nav1.7 channel activation in the depolarizing direction by 31.1 mV [33]. ProTx-II is a typical gating modifier toxin and can inhibit multiple sodium channel subtypes (Na_v_1.1–1.8), but preferentially inhibits hNa_v_1.7 [34]. Moreover, ProTx-II is not efficacious in rodent models of acute and inflammatory pain [35]. However, the mechanism of action of HpTx3 is somewhat similar to those of HwTx-IV [20], HnTx-III [36], HnTx-IV [37], etc.; these toxins do not alter the I-V relationship for the Na_v_1.7 and induce minor (less than 5 mV) alterations in the voltage-dependence of its activation and steady-state inactivation [7]. Based on the mechanism of action, the Na_v_1.7-targeting peptide toxins are classified as pore blockers and/or gating modifiers [38]. In fact, pure pore blockers or pure gating modifier toxins in nature are rare. In many cases the modes of action, namely “pore blocker” or “gating modifier”, coexist with different potencies [39]. According to the action characteristics of HpTx3, we propose that this toxin acts as both a pore blocker and a gating modifier.

In order to localize the binding sites of HpTx3 on Na_v_1.7 for further understanding the mechanism of action of HpTx3, we mutated the Na_v_1.7 by using the domains or domain extracellular loop of Na_v_1.8, on which 10 µM HpTx3 showed no effect (Figure 3), to replace the counterpart sequences of the Na_v_1.7. As a result, the currents of chimeras Na_v_1.7/1.8DIIS3b–S4 and Na_v_1.7/1.8DIV could not be inhibited by 1 μM HpTx3 (Figure 5); the amino acid residue D816 in the DIIS3b–S4 sequence was shown to be a key residue for HpTx3 binding. It is worth mentioning that Na_v_1.6 also has D residue in its S3b–S4 sequence. However, the inhibitory effect of HpTx3 on the Na_v_1.6 was weaker than that on Na_v_1.7, suggesting that there were other factors affecting the binding of HpTx3 to the ion channel. Our experimental results demonstrated that DIIS3b–S4 and DIV of Na_v_1.7 participated in HpTx3 binding to Na_v_1.7, and the effect of HpTx3 on Na_v_1.7 involves the synergistic action of domain II and domain IV. We propose that the interaction of HpTx3 with DII and DIV of Na_v_1.7 maybe at least partially contributes to the shift of the activation and steady-state inactivation curves (Figure 4), because the neurotoxins acting on Domain II of Na_v_ channels (site 4 toxins) often affect the activation of targeted channels, and DIV-interacting (site 3) toxins affect the inactivation kinetics of the channels [40,41]. Such a cooperative action of the domain II and IV of Na_v_ channels has also been reported previously. For example, during the interaction of RTX-VII, a toxin from the venom of the *Macrothele raveni* spider, with Na_v_1.3, the domains II and IV of the Na_v_1.3 cooperatively contribute to the generation of the persistent current in Na_v_1.3 [42]. In fact, many Na_v_-targeted toxins have been reported to have more than one binding site on Na_v_ channels. β-scorpion toxins, for instance, like α-scorpion toxin [43,44], have additional interaction points on the extracellular domains of Na_v_ channels in addition to their binding site on the domain IIS3–S4 extracellular loop sequence [40].

In view of the potent and selective action of HpTx3 on Na_v_1.7, we investigated the pharmacological implications of HpTx3 in analgesia, using five different mouse pain models induced by formalin, acetic acid, hot plate, spared nerve injury, and complete Freund’s adjuvant, respectively.

The formalin pain model is an effective model commonly used in pain and analgesic research [45]. In this model, formalin induces distinct biphasic nociceptive responses. The phase I responses were thought to be produced by direct activation of nociceptive neurons by formalin, and the phase II responses were caused by the formalin-induced inflammation [21,22]. Our experimental results demonstrated that HpTx3 could efficiently inhibit the nociceptive responses in both of the two phases, suggesting that the toxin not only suppressed the pain-like behaviors induced by nociceptive nerve activation, but also inhibited those caused by inflammation induced by tissue injury. Furthermore, the effects of HpTx3 are somewhat similar to those of the centrally acting drugs that generally inhibit both of two phases equally [46]. The findings that HpTx3 and morphine, a drug that can be used as the positive control in studying central analgesic activities [47], exhibited similar analgesic effects support the conclusion. The experimental results suggest that HpTx3 can affect some central nervous system-mediated behaviors. A drug may exert such an effect after crossing the blood–brain barrier and/or by producing active components that are transported into the central nervous system [48]. However, how the HpTx3 affects the nervous system-mediated behaviors needs further investigation. Considering that HpTx3 could inhibit the pain-like behaviors caused by chemical or thermal stimuli in the mouse pain models prepared with induction of formalin, acetic acid, complete Freund’s adjuvant, and hot plate, we speculate that the analgesic action of HpTx3 might involve blocking the release of prostaglandins and some other inflammation-inducing substances, because these inflammation pain models could induce pain-like behavior by releasing these bioactive components to excite nociceptive nerve endings [47]. 

In our present study, a sciatic nerve spared nerve injury (SNI) pain model was also developed to evaluate the analgesic effect of HpTx3. In this SNI model, the SNI-operated mice displayed rapid, strong, and persistent hypersensitivity to mechanical stimuli in the territory of the spared sural nerve, and thus are suitable to be used for detecting the mechanisms involved in mechanical pain [49]. As a result, HpTx3 was shown to display a strong analgesic effect in the SNI model, increasing the paw withdrawal threshold of the mice in a dose-dependent manner. Taken together, HpTx3 showed potent analgesic effects in all the used mouse pain models, indicating that the toxin has inhibitory action on acute, inflammatory, and neuropathic pains.

Of the nine Na_v_ channel subtypes, Na_v_1.3 and Na_v_ 1.7–1.9 are widely considered to be associated with nociception and play essential roles in the pain pathway [50]. However, our experiments demonstrated that 1 μM HpTx3 has no effects on Na_v_1.3 and even 10 μM HpTx3 does not affect the currents of Na_v_1.8 and Na_v_1.9. Although Na_v_1.6 was recently reported to be associated with local inflammation and neuropathic pain [51,52], the Na_v_ 1.6 is primarily expressed in the central nervous system and mature nodes of Ranvier in the peripheral nervous system [53], and HpTx3 preferentially inhibits Na_v_1.7 with at least seven-fold selectivity against Na_v_1.6. Therefore, in the HpTx3-caused analgesia, Na_v_1.7 was the mediator and the role of Na_v_1.6, if any, is limited. In our present study, due to the limitation of experimental conditions we did not detect the effect of HpTx3 on Na_v_1.1. However, although Na_v_1.1 has been demonstrated by Osteen et al. [54] to be also involved in pain, the role of the Na_v_1.1 in the HpTx3-mediated analgesia are speculated to be relatively minor; this is because Na_v_1.1 transcripts are primarily expressed by medium-diameter sensory neurons (only constituting 35% of all neurons within the DRG), and the Na_v_1.1-mediated pain behaviors are not associated with neurogenic inflammation and thermal stimulation [54], whereas the Na_v_1.7 has even extensive distribution, including being expressed in both large and small diameter DRG neurons [9], and shows efficient analgesic effects in multiple mouse pain models, including inflammation model and that induced by heat plate-produced thermal stimulation. Therefore, like Na_v_1.6, we expect the effect of Na_v_1.1 for HpTx3-induced analgesia to be relatively small.

In addition, it should be mentioned that the HpTx3 has been demonstrated to inhibit K_v_4 channels, particularly K_v_4.2 [17,24,25], and the K_v_4 channels are also involved with pain transmission. K_v_4 channels are expressed predominately in the somata of small and large diameter nociceptors and the dorsal horn of the spinal cord, and all three K_v_4 mRNA isoforms (K_v_4.1–4.3) are expressed in whole DRG preparations [55,56,57,58]. Overexpression of various components of the K_v_4 complex attenuated DGR excitability and pain phenotypes of animals [59]. The drugs that can increase A-type K_v_ currents reverse the pain phenotype [58]. On the contrary, reduced expression of the K_v_4 channels and knockdown of any component of the K_v_4 channel complex in primary neurons induce mechanical hypersensitivity, a major symptom of neuropathic pain [60,61]. Oxaliplatin, a chemotherapy drug, increases nociceptive neuron excitability to result in neuropathic pain in orofacial and other regions in patients, and the down-regulation of K_v_4.3 channels and I_A_ currents may be an underlying mechanism of oxalipaltin-induced orofacial neuropatic pain [62]. Peptide toxin Ts8 from the venom of the *Tityus serrulatus* (Ts) scorpion inhibited the K_v_4.2 channel, decreasing the mechanism nociception threshold and inducing obvious nociception [63]. These observations indicate that the levels of K_v_4 proteins and A-type currents are reversely related to the nociceptive neuron excitability. That is to say, HpTx3 inhibition of K_v_4 channels [17,24,25] might result in hyperalgesia, an effect contrary to analgesia caused by the HpTx3 inhibition of Na_v_1.7. Nevertheless, in our present study, HpTx3 used in several pain models showed potent analgesic activity, suggesting that the analgesic effect of HpTx3 overwhelmed the hyperalgesia it caused. This result may be explained at least partially by the fact that in the pain models the level of K_v_4 channels is usually decreased [56,61,64,65]. Till now, only one model showed that the expression of K_v_4 channels was increased following injury [58]. In addition, the major K^+^ channel α subunit responsible for transient outward K^+^ current (*I*_to_) in rat is K_v_4.2 [66], whereas the rat DRG primarily expresses K_v_4.1 and K_v_4.3 of K_v_4 channels [59], which could provide an additional explanation for the cause that the analgesic effect of HpTx3 masked the hypersensitivity caused by HpTx3 inhibition of Kv4 channels. JzTx-V has similar characteristics as the toxin can inhibit K_v_ channels including K_v_4.2 [67], Kv4.3 [68], and Na_v_1.7 channels [69], and it also shows a potent analgesic effect in the pain models [70].

## 4. Conclusions

The peptidic neurotoxin HpTx3 from the venom of the spider species *Heteropoda venatoria* was newly found to have a selective inhibitory action on Na_v_1.7. With the characteristics of both pore-blocker and gate-modifier, HpTx3 does not alter the current–voltage (I–V) relationship of the Na_v_1.7 channel but makes minor alternation in the voltage-dependence of activation and steady-state inactivation of Na_v_1.7 by interacting with the S3b–S4 sequence in domain II and domain IV of Na_v_1.7. During the interaction of HpTx3 with the S3b–S4 sequence of Na_v_1.7, D816 plays a key role, whereas the E818 only plays a minor role. HpTx3 displayed a potent analgesic effect in five mouse pain models prepared based on different nociceptive mechanisms in a dose-dependent manner, suggesting that acute, inflammatory, and neuropathic pains were all attenuated by the toxin. Generally, HpTx3 at doses of ≥1 mg/kg could produce the analgesic effect comparable to that of 1 mg/kg morphine. The experimental results suggest that HpTx3 can act not only as a molecular probe in the researches on ion channel functions and pain mechanisms, but also as a lead molecule in the development of the drugs that treat Na_v_1.7 channel-related pain.

## 5. Materials and Methods 

### 5.1. Purification and Identification of HpTx3

The venom of the spider species *Heteropoda venatoria* was collected by electrical stimulation, lyophilized and stored at −20 °C before use [71,72]. For separation, the crude venom was dissolved in distilled H_2_O to a final concentration of about 1 mg/mL and then subjected to semipreparative RP-HPLC (C18 column, 10 µm, 10 mm × 250 mm, Welch Materials, Inc., Shanghai, China). The loaded venom components were eluted with a linear acetonitrile gradient (20–55% acetonitrile/0.1% TFA in 45 min) at a flow rate of 3.0 mL/min. The peak containing HpTx3 [17] was collected, lyophilized, and further purified to homogeneity by analytical RP-HPLC (C18 column, 5 μm, 4.6 mm × 250 mm, Phenomenex Inc., Torrance, CA, USA). The molecular weight of the toxin was determined with an ESI mass spectrometer (6540Q-TOF, Agilent Technologies, Santa Clara, CA, USA).

### 5.2. Plasmid Construction and Transient Transfection

All the used Na_v_ channel subtype clones (Na_v_1.2–Na_v_1.9) and β subunit clones were preserved in our laboratory. cDNAs encoding rat Na_v_1.2, rat Na_v_1.3, human Na_v_1.5, and mouse Na_v_1.6 were subcloned into the vector pcDNA3.1; cDNA encoding rat Na_v_1.4 was subcloned into the vector pRGB4; cDNA encoding human Na_v_1.7 was subcloned into the vector pcDNA3.1-mod; cDNAs encoding rat Na_v_1.8 and human Na_v_1.9 were subcloned into the vectors pCMV-HA and pEGFP-N1, respectively. For localizing the binding sites of HpTx3 on the Na_v_1.7 and therefore probing the its mechanism of action, several chimeras were constructed with a recombination strategy by replacing the extracellular loop S3b–S4 in domain II, domain III, and domain IV of Na_v_1.7 with the counterpart sequences of Na_v_1.8, respectively, using the methods described [42,73,74]. As a result, three chimeras were prepared, namely, Na_v_1.7/1.8DIIS3b–S4, Na_v_1.7/1.8DIII, and Na_v_1.7/1.8 DIV. Moreover, the amino acid residues D816 and E818 in the S3b–S4 sequence of Na_v_1.7 were mutated into N (Na_v_1.7 D816N) and Q (Na_v_1.7 E816Q), respectively.

For heterologous expression, the vector plasmids containing wild-type (WT) Na_v_1.2–Na_v_1.7 and mutant plasmids were transiently transfected into HEK293T cells (China Center for Type Culture Collection, Wuhan, China) using Lipofectamine 2000 (Invitrogen, Carlsbad, CA, USA) according to the manufacturer’s instructions. The vectors containing rNa_v_1.8 and hNa_v_1.9 were transiently transfected into the neuron-like ND7/23 cells (Spring Bioscience, Shanghai, China), which were more suitable for their heterologous expression [75,76]. In addition, the plasmids β1- and β2-eGFP, which encode the β1-subunit and β2-subunit, respectively, were co-transfected with those encoding WT Na_v_1.7 and its mutants into the HEK293T cells. HEK293T and ND7/23 cells were maintained at 37 °C in a humidified 5% CO_2_ incubator in Dulbecco’s modified Eagle’s medium (DMEM) supplemented with 10% fetal bovine serum, 2 mM L-glutamine, 100 U/mL penicillin, and 100 μg/mL streptomycin. When the cells were grown to about 90% confluence, transfection was performed with the vector plasmids. The cells with green fluorescence were selected for whole-cell patch-clamp analysis 24 h after transfection.

### 5.3. Whole-Cell Patch-Clamp Analysis

The whole-cell patch-clamp technique was employed to analyze the effects of HpTx3 on Na_v_ channel subtypes in the transfected HEK293T and ND7/23 cells using an EPC-10 USB patch-clamp amplifier (HEKA, Elektronik, Lambrecht, Germany). The suction pipettes with DC resistance of 2.0–3.0 MΩ were fabricated from borosilicate glass capillary tubes (VWR micropipettes; VWR Co., West Chester, PA, USA) using a two-step vertical microelectrode puller (PC-10, Narishige Co., Ltd., Tokyo, Japan). For recording the Na^+^ currents, the suction pipettes were filled with an intracellular solution of the following composition: 140 mM CsCl, 35 mM NaCl, 105 mM CsF, 10 nM EGTA (ethylene glycol-bis (2-aminoethyl ether)-N, N, N’, N’-tetraacetic acid), 10 nM HEPES (4-(2-hydroxyethyl)-1-piperazineethanesulfonic acid), pH7.4. The bath solution was composed of 140 mM NaCl, 2 mM CaCl_2_, 1 mM MgCl_2_, 5 mM KCl, 20 mM HEPES, and 10 mM glucose, pH 7.4. The experiments were conducted at room temperature (20–25 °C). All chemicals were products of Sigma-Aldrich (St. Louis, MO, USA) and were dissolved in sterile double-distilled H_2_O. Data were collected with the PatchMaster software in the HEKA EPC-10 USB patch-clamp system (HEKA Elektronik, Lambrecht, Germany) and analyzed by the software Igo Pro-6.00, Prism (GraphPad Software, La Jolla, CA, USA), Sigmaplot 10.0 (Systat Software, Inc., San Jose, CA, USA), and OriginPro.

In whole-cell patch-clamp analysis, to induce ion channel currents, a 50-ms depolarization ranging from a holding potential of −80 mV to −10 mV was used for Na_v_1.2–Na_v_1.7, −100 mV to +20 mV, and −120 mV to −40 mV for Na_v_1.9. When the currents of Na_v_1.8 and Na_v_1.9 were measured, 1 µM TTX was added to inhibit the TTX-sensitive currents. To assess the current–voltage (I–V) relationship of Na_v_1.7, the currents were induced by a series of 50-ms step depolarization potentials ranging from −80 mV to +80 mV with a 10-mV increment at 5 s intervals. The voltage-dependence of activation was assessed with a series of test potentials in the range −80 mV to +40 mV from a holding potential of −100 mV with 10-mV increment. For detection of the voltage-dependence of inactivation, the currents were induced by a 50-ms depolarizing potential of −10 mV from various prepulse potentials for 500 ms that ranged from −120 mV to −20 mV with a 10-mV increment.

### 5.4. In Vivo Analgesic Activity Assay

#### 5.4.1. Animals

Healthy female C57BL/6 mice (weighting 18–20 g) that were used for analgesic activity assay were obtained from the Experimental Animal Center of SLac-kinda (Changsha, China) and were randomly assigned to five groups, each group generally containing 8 mice. One group was used as the blank control, one group as the positive control treated with 1 mg/kg morphine, and three groups as the test groups treated with different concentrations of HpTx3 (0.2, 1, and 5 mg/kg), respectively. The mice were maintained at a temperature of 20–25 °C and under a 12 h light/12 h dark cycle, with free access to food and water. The ethical approval for the in vivo animal experiments was obtained from the Animal Care and Use Committee of the Hunan Province Animal Management Office. (The approval code: 219/2019).

#### 5.4.2. Formalin-Induced Paw Licking

The changes in the formalin-induced nociceptive reaction in mice was used to assess the in vivo effect of HpTx3 on Na_v_1.7-mediated pain [74,77,78]. Paw licking, a pain-related behavior, was induced by subcutaneous intraplantar injection of formalin. The mice were intramuscularly injected into the inner thigh of the right hind limb with 100 μL 0.9% saline, morphine (1 mg/kg), or HpTx3 (0.2, 1.0, and 5.0 mg/kg), respectively, followed by subcutaneous intraplantar injection of 20 μL formalin (10%) at the right hind paw. The time that was spent to lick the injected paw by each mouse during phase I (0–10 min after injection) and phase II (15–35 min after injection) was separately recorded with a digital stopwatch. The nociceptive behavior attenuation by HpTx3 was evaluated by comparison of the paw licking times of the mice in each group.

#### 5.4.3. Acetic Acid-Induced Abdominal Writhing

Abdominal writhing responses were induced by intraperitoneal injection (i.p.) of acetic acid to C57BL/6 mice according to a previous study [10]. The mice were fasted for 12 h but were allowed to drink water freely before being used in analgesic experiments. The mice in the test groups were intraperitoneally injected with HpTx3 (0.2, 1.0, or 5.0 mg/kg) while the mice in the control groups received an equal volume of 0.9% saline. Fifteen-five minutes later, 100 μL of 1% (*v*/*v*) acetic acid were intraperitoneally injected. Then the abdominal writhing responses were observed and the writhing number of each mouse was counted for 30 min. Each bend, indent, lift, or stretch was counted as one “writhing”.

#### 5.4.4. Complete Freund’s Adjuvant (CFA)-Induced Hyperalgesia

According to the previously described methods with some modifications [34,78], a complete Freund’s adjuvant (CFA) was administered undiluted in a volume of 20 μL by intraplantar injection at the right hind paw of four-week-old C57BL/6 mice. Paw withdrawal thresholds to mechanical stimulus in the inflamed paw were determined 24 h after the injection. HpTx3 (0.2, 1 and 5 mg/kg) and morphine (1 mg/kg) were dissolved in 0.9% saline and administered in a volume of 20 μL by intramuscular injection into the inner thigh of the right hind paw 30 min prior to the withdrawal threshold determination. The mechanical paw withdrawal thresholds were determined at 0.5, 1, 2, 3, and 4 h after the injection by using a set of calibrated Von Fery filaments with forces from 0.4 to 2.0 g (Stoelting Co., Wood Dale, IL, USA) according to the previously described method with some modifications [79]. At each time point, the plantar surface of mice was stimulated by the Von Frey filament with sufficient force to cause slight bucking against the paw, holding for up to approximately 6–8 s. The measurement was performed once every 10 s within a period of 100 s. When the number of paw withdrawal was less than 6, a next filament with increasing force was used. The smallest force (g) of the used Von Frey filaments that made the number of paw withdrawal be greater than 5 was defined as the mechanical paw withdrawal threshold at that time point.

#### 5.4.5. Hot Plate Test

Hot plate test was conducted according to the method described [47]. The hot plate test apparatus (model YSL-21, Jinan, China) was placed in a quiet room, and the temperature was set at 55 ± 1 °C. After each mouse was placed at the hot plate, its nociceptive responses (hind-paw licking or jumping) and nociceptive threshold (paw withdrawal latency) were observed and recorded. A total of 0.9% Saline, morphine (1 mg/kg), and HpTx3 (0.2, 1, and 5 mg/kg) were separately intraperitoneally injected into the mice and the paw withdrawal latency was recorded at 0.5, 1.0, 1.5, and 2.0 h after the injection. The mice displaying abnormal baseline paw withdrawals (shorter than 5 s and longer than 30 s) were excluded from the test.

#### 5.4.6. Spared Nerve Injury (SNI)

When the SNI model was prepared, the surgery was performed according to the method of Bourquin et al. [49]. Briefly, under 1.5–2.5% isoflurane general anesthesia, the left hindlimb was fixed in a lateral position. Incision was made at mid-thigh level and a section was made through the biceps femoris in the direction of point of origin of the vascular structure to expose the three peripheral branches of the sciatic nerve. Both common peroneal and tibial nerves were ligated with a 6.0 silk thread, with the sural nerve being carefully preserved. The muscle and skin were closed in two distinct layers with the 6.0 thread. The sham surgery was performed on the control mice in the same way, without any nerve ligation. Mechanical sensitivity was recorded eighteen days after the surgery. The mice in control (sham-operated) group and test (SNI) groups each were intraperitonealy injected with 10 µL of physiological saline or HpTx3 (0.2, 1, and 5 mg/kg) in physiological saline, respectively. The plantar side of the paw ipsilateral to the surgery was stimulated with calibrated Von Frey monofilaments with forces from 0.4 to 2.0 g (Stoelting Co., Wood Dale, IL, USA) and the mechanical paw withdrawal threshold was determined as described in a complete Freund’s adjuvant (CFA) model.

### 5.5. In Vivo Toxicity and the Effect of HpTx3 on the hERG Channel

For detecting the potential in vivo toxicity of HpTx3, eight C57BL/6 mice were each intraperitoneally injected with HpTx3 at a dose of 20 mg/kg, which was 4-fold greater than the maximum dose (5 mg/kg) and 100-fold greater than the minimum dose (0.2 mg/kg) that were used in analgesic experiments.

The effect of HpTx3 on the hERG channel expressed in HEK293T cells was assayed using the whole cell patch-clamp technique according to the previous method with some modifications [80]. The bathing solution contained (in mM): HEPES 10, MgCl_2_ 1, CaCl_2_ 25, KCl 5, NaCl 50 (pH 7.4, adjusted with 1 M NaOH). The suction pipettes solution contained (in mM): MgCl_2_ 2.5, KCl 140, EGTA 11, HEPES 10 (pH 7.2, adjusted with KOH). The osmotic pressure of the two solutions was adjusted to 280 ± 5 mOsm/L with sucrose. The currents of the hERG channel were induced at −40 mV after a test potential of +30 mV from a holding potential of −80 mV.

### 5.6. Data Analysis

The acquired electrophysiological data were analyzed with SigmaPlot 10 software (Sigma, St. Louis, MO, USA) and Prism 5 (GraphPad Software, San Diego, CA, USA). Concentration–response curves were fitted by the SigmaPlot sigmoidal equation as follows: y = a/(1 + exp(−(x − IC_50_)/b)), in which IC_50_ is the concentration of toxin at half-maximal efficacy, and a and b are the constants. The conductance (G) at each test potential (V) was calculated according to the equation G = I/V − V_rev_, where *V*_rev_ is the reversal potential, V is the test potential, and I is the current amplitude. Normalized peak conductance was fitted with the Boltzmann equation G/G_max_ = 1/{1 + exp[(V_1/2_ − V)/κ]}, where G_max_ is the maximum conductance, V_1/2_ is the membrane potential of half-maximal activation, and κ is the slope factor. Peak inward currents from steady-state inactivation were normalized by the maximum current amplitude and fitted with a Boltzmann equation: I/I_max_ = 1/{1 + exp[(V − V_1/2_)/κ}, where V represents the inactivating prepulse potential, V_1/2_ is the membrane potential of half-maximal inactivation, and κ is the slope factor.

Statistical analyses were performed using paired Student’s *t*-test or ANOVA with paired comparisons. All data are presented as mean ± SD, and statistical significance was accepted at *p* < 0.05.
